# Factors determining speed management during distracted driving (WhatsApp messaging)

**DOI:** 10.1038/s41598-020-70288-4

**Published:** 2020-08-06

**Authors:** Sonia Ortiz-Peregrina, Oscar Oviedo-Trespalacios, Carolina Ortiz, Miriam Casares-López, Carlos Salas, Rosario G. Anera

**Affiliations:** 1 Laboratory of Vision Sciences and Applications, Department of Optics, University of Granada, Edificio Mecenas, Av. Fuentenueva s/n, 18071 Granada, Spain; 2 Centre for Accident Research and Road Safety-Queensland (CARRS-Q), Queensland University of Technology (QUT), Brisbane, 4059 Australia

**Keywords:** Engineering, Optics and photonics

## Abstract

The objective of this work was to investigate self-regulation behaviours, particularly speed management, under distracted conditions due to WhatsApp use. We also studied the influence of different environments and driver characteristics, introducing visual status as one of them. Seventy-five drivers were evaluated in a simulator study involving two test sessions under baseline and texting conditions. A cluster analysis was used to identify two groups with different visual capacity .Lastly, possible predictors of speed management were studied developing a generalised linear mixed model. Our results show that drivers reduced their speeds in the presence of more demanding driving conditions; while replying to a WhatsApp message, on curved road segments and when parked cars are present. Driving speed also correlated with driver characteristics such as age or dual task experience and human factors such as self-perceived risk. Finally, although there were significant differences in visual capacity between the two groups identified, the model did not identify visual capacity membership as a significant predictor of speed management. This study could provide a better understanding of the mechanisms drivers use when WhatsApp messaging and which environments and driver conditions influence how speed is managed.

## Introduction

Driving is a highly demanding task; drivers must manage their cognitive, physical and visual skills continuously in order to operate the vehicle. Distractions easily interrupt this task while drivers must manage the distribution of their resources to ensure safe driving. There are different sources of distraction, from the vehicle itself^1^ to the driving environment^1,2^. In Spain, driver distraction was the cause of 32% of all accidents recorded in 2018, with mobile phone use standing out as the main source of distraction^3^. Over 40% of Spanish drivers admit to sending text messages while driving^4^. High percentages have also been reported in other countries such as the United States (~ 60%)^5^ or Australia (33.5%)^6^. Although texting while driving is banned, this trend is expected to continue or even increase in the coming years This is due to the emergence of smartphones and instant messaging applications such as WhatsApp, which have assumed a major role in our daily communications, offering users much greater dynamism compared to SMS messages (Short Message Service)^7^.

Research has repeatedly highlighted the negative effects of texting on driving performance^8-10^. This driving behaviour doubles the risk of an accident^11^, despite the fact that drivers typically self-regulate their driving when distracted. Self-regulation is a dynamic strategy that drivers use to manage the demands on the resources they require to control the vehicle and perform the secondary task, prioritising the former to minimise the safety risk as much as possible^12^. Self-regulation while distracted includes operations such as paying less attention to the secondary task^12^, over correcting the vehicle’s position^9,13^, and overcorrecting or reducing speed^10,12,13^. Speed reduction is a behaviour commonly observed in all distraction types^8,10,14,15^ because of the difficulties drivers experience in their performance^16^.

However, the management of resources while distracted and the consequent behaviours seem to be influenced by other factors. The task–capability interface model developed by Fuller et al. (2008)^17^ indicated that speed management is the result of combining influences related to the vehicle, the environment and the driver. With respect to the environment, some studies have shown that people reduce their speeds in function of certain road characteristics^6,18,19^, in heavy traffic^20,21^, and in situations with more visual information such as urban roads^10,19^. Similarly, some evidence has suggested that driver characteristics such as age, sex or other personal traits have an impact on speed management^1,19^.

While driving, we have to carry out precise searches in environments cluttered with visual information in order to produce a rapid and effective response, which may be vital for safety reasons. We also self-regulate vehicle speed according to visual information from the environment; for example, reducing speed to comply with road signs, in anticipation of a potential hazard or to adapt to current traffic conditions. Drivers with a deteriorated visual capacity may find it even harder to detect visual information in complex scenarios, e.g., with considerable amounts of visual clutter^22^. Ageing promotes a natural decrease in visual function^23-25^ and this is significant even when visual acuity is much higher than the minimum required for driving. In fact, different studies have shown that among older drivers, visual impairment is one of the leading causes of driver behaviour modification, limiting their exposure to situations perceived as more challenging, such as adverse meteorological conditions, heavy traffic or high speeds^26,27^. A worse visual status could imply longer periods of distraction from the road when texting, leading to a greater speed reduction as a compensatory mechanism. Both driving and typing WhatsApp messages are strongly dependent visual tasks, so worse vision can be expected to have an influence on speed adaptation mechanisms, but this issue has not yet been investigated in previous studies.

Thus, the aim of this study was to investigate self-regulation behaviours, and more specifically speed management, when distracted due to WhatsApp use. Using WhatsApp while operating a moving vehicle involves visual–manual interactions, which is now a major concern in terms of road safety. Therefore, we examined the influence of different environments and driver characteristics by introducing visual status as one of the factors that could affect how distraction is managed while at the wheel.

## Methods

### Participants

Seventy-five drivers (19–68 years) were recruited for the study. All were in good general health and did not have any eye diseases. Participants were required to have a binocular visual acuity of 20/40 or better, the legal level for driving in Spain. They must have had a valid driving license for at least one year and driven at least 1000 km in the last year. Likewise, participants were required to be experienced WhatsApp users (≥ 30 WhatsApp messages per day). Table 1 shows the demographic characteristics of the drivers involved in the study.

**Table 1:** Sociodemographic characteristics of the sample (continuous variable age is shown as mean ± SD).

Sociodemographic characteristics		Mean (± SD)/N (%)
Age (years)		38.7 (± 15.0)
**Gender**		
Male		53 (70.7)
Female		22 (29.3)
**Experience texting while driving**		
0	Never	45 (60)
1	1–2 times a year	6 (8)
2	1–2 times a month	10 (13.3)
3	1–2 times a week	8 (10.7)
4	Daily	6 (8)
**Self-perceived increase in risk while texting**		
0	None	0 (0)
1	Slight	0 (0)
2	Somewhat	1 (1.3)
3	Quite a lot	13 (17.3)
4	A lot	61 (81.4)

The study was approved by the University of Granada Human Research Ethics Committee (180/CEIH/2016). Prior to the testing sessions, all subjects signed the informed consent form in accordance with the Declaration of Helsinki.

### Visual assessment

#### Visual acuity

Visual acuity (VA), or the ability to resolve detail, is a standardised visual test used by licensing authorities worldwide in driver screening procedures. In our study, VA was measured with the POLA VistaVision Visual Acuity Chart at 5.5 m (logMAR scale) employing Snellen letters.

#### Contrast sensitivity

Contrast sensitivity is a visual test used to study the eye’s ability to distinguish between an object and the background, and not only size. Contrast sensitivity function (CSF) was obtained experimentally by measuring the contrast threshold (i.e., the contrast required to reliably perceive a visual target on a uniform background). Thus, CSF was calculated from the inverse of the contrast threshold as a function of spatial frequency. We used the CSV-1000 test (VectorVision, Ohio, USA) at the recommended viewing distance (2.5 m) and expressed in log units to measure this clinical parameter. More details of this visual test are provided elsewhere^28^.

The two visual tests were performed binocularly, with participants wearing their normal optical correction used when driving.

#### Driving simulator: road scenarios

The virtual driving environment used in this study was generated on three high-definition 27″ screens, with a resolution of 1920 × 1080 pixels and a 180° field of view.

It was employed a fixed-base driving simulator (Logitech G27 Racing Wheel, Logitech International S.A., Lausanne, Switzerland) and all driving routes were generated with SIMAX DRIVING SIMULATOR v4.0.8 BETA (SimaxVirt S.L., Pamplona, Spain) software.

**Figure 1:**
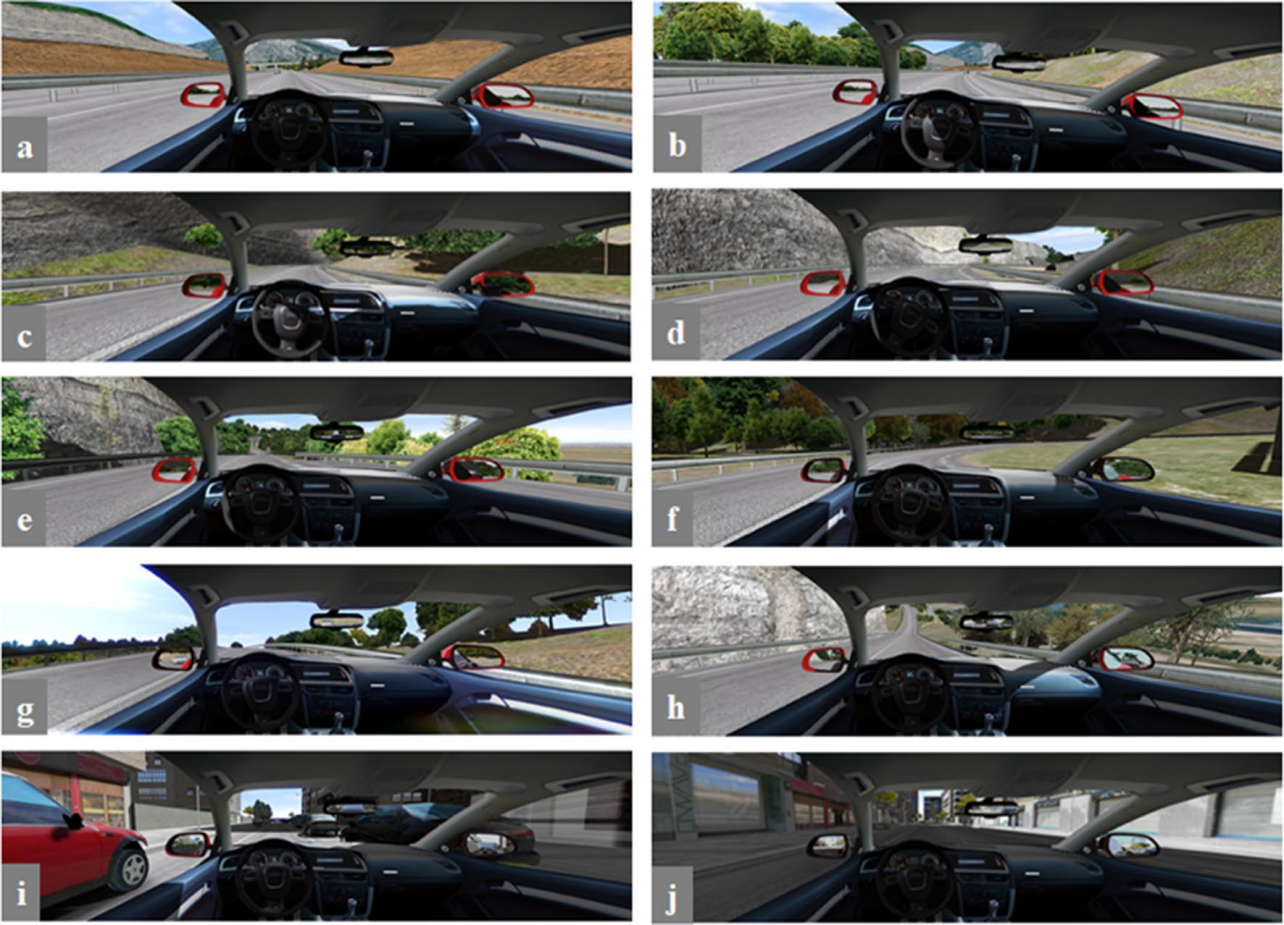
Screenshot of the different driving scenarios selected for the analysis (a-j correspond to scenarios 1–10).

The route was approximately 12.5 km long and took about 15 min to complete. It included three different main road types, similar to those which can be found on the Spanish road network: dual carriageway, mountain road and an inner-city circuit. From these three road types we choose 10 different scenarios for analysis with varying combinations of road geometry and traffic complexity (Table 2, Fig. 1). Road types involved different speed limits. Traffic complexity included the presence of oncoming cars or other vehicles in the same direction. Road geometry refers to the road layout (straight, slight bend or sharp bend) and the presence and type of slope (no slope, gentle, steep, ascending or descending slope). The inner-city road type also featured parked cars around the driver.

**Table 2:** Characteristics of the different driving scenarios selected for the analysis.

Scenario	Road type	Speed limit (kph)	Road geometry and traffic complexity			
			Other traffic	Road geometry		Parked cars around
				Road layout	Slope	
1	Dual carriageway	120	Same direction	Straight	No	No
2	Dual carriageway	120	Same direction	Slight bend	No	No
3	Mountain road	90	Oncoming Same direction	Straight	Gentle/ascending	No
4	Mountain road	90	Oncoming Same direction	Sharp bend	Gentle/ascending	No
5	Mountain road	40	Oncoming Same direction	Straight	Gentle/ascending	No
6	Mountain road	40	Oncoming Same direction	Sharp bend	Gentle/ascending	No
7	Mountain road	90	Oncoming Same direction	Straight	Steep/ascending	No
8	Mountain road	90	Oncoming Same direction	Straight	Steep/ descending	No
9	City	50	Same direction	Straight	No	Yes
10	City	50	Same direction	Straight	No	No

For driving performance data analysis, we selected a representative length of 100 m along each driving scenario. This type of analysis has been used previously^19^, as it means both traffic conditions and road geometry are as uniform as possible throughout the section being analysed, thus guaranteeing that driving performance is studied under specific conditions. Furthermore, there must be sufficient separation between the various sections with different characteristics that are used in the analysis. This ensured the sections did not have an influence on each other because drivers were still in the process of adapting their driving to each new scenario.

#### Experimental procedure

All participants received at least two training sessions of 15 min before the experiment, with a 1-week washout period between them. Then, they were tested in two different sessions to measure driving under baseline and texting conditions.

In the texting condition, participants received six WhatsApp messages, with five short general knowledge questions and one simple mathematical problem. They were instructed to answer these questions in a similar manner as occurs in actual driving, that is, prioritising the driving task. All messages were of a similar length (30–55 characters) and sent at specific points along the route that were strategically selected so drivers could be observed performing the dual task in the 10 scenarios selected for data analysis. Participants drove with the smartphone held by a support located to the right of the steering wheel. They used their own smartphones to ensure they were familiar with its operation.

### Data analysis

#### Speed management

Speed management was analysed for all the scenarios and both driving conditions (baseline and texting). To this end, we calculated how much the participants’ speed deviated from the displayed limit (driving speed—speed limit). Therefore, negative values of deviation from the speed limit means the driver went slower than the limit, which suggests an increase in safety^29^.

#### Data analysis and statistical procedures

Data analysis involved two main phases. Firstly, a two-step cluster analysis method was chosen to classify participants into different categories of visual status. This technique assigns participants to a cluster by minimising within-cluster variance and maximising between-cluster variance. The number of clusters is selected using the Akaike information criterion (AIC). The second phase of the study analysed the drivers’ behaviour on different road geometries using a generalised linear mixed model (GLMM) with repeated measures.

The GLMM can be represented as follows^13,30^:

1 $$g(\mu_{ij} ) = \alpha + {\mathbf{X}}_{i}^{{\prime }} {{\varvec{\upbeta}}} + {\mathbf{Y}}_{i}^{{\prime }} {{\varvec{\upgamma}}} + {\mathbf{Z}}_{j}^{{\prime }} {{\varvec{\uplambda}}}$$

where *g* is the Gaussian link function, **α** is the intercept, **β**, **γ** and **λ** are estimated coefficients of the independent variables. ${\mathbf{X}}_{i}$ is a vector of driver characteristic variables (age, gender, visual status, experience texting while driving and self-perceived risk), ${\mathbf{Y}}_{i}$ is a vector of the driving conditions variable (baseline or texting), and ${\mathbf{Z}}_{j}$ is a vector of variables used to describe the road environment (scenarios 1–10). Coefficients of the link function in the GLMM are estimated from the following equation^13,30^:

2 $$S(\beta ) = \sum\limits_{i = 1}^{k} {\frac{{\partial \mu_{i}^{{\prime }} }}{\partial \beta }} V_{i}^{ - 1} (SA_{i} - \mu_{i} (\beta )) = 0$$

where $V_{i}$ corresponds to an estimation of the covariance matrix of $SA_{i}$ specified as $V_{i} = \phi A_{i}^{1/2} R_{i} (\rho )A_{i}^{1/2}$. Where $A_{i}$ is an $n_{i} \times n_{i}$ diagonal matrix with $v(\mu_{ij} )$ as the *j*th diagonal element. $V_{i}$ varies between drivers, but it can be assumed to have the same form for all drivers. $R_{i} (\rho )$ is an $n_{i} \times n_{i}$ working correlation matrix specified as $\rho$. Constant correlations between any two observations for a given driver are defined as:

3 $$Corr(SA_{ij} ,SA_{il} )\left\{ {\begin{array}{*{20}l} 1 \hfill & {j = l} \hfill \\ \rho \hfill & {j \ne l} \hfill \\ \end{array} e.g.R_{4x4} } \right. = \left[ {\begin{array}{*{20}c} 1 & \rho & \rho & \rho \\ \rho & 1 & \rho & \rho \\ \rho & \rho & 1 & \rho \\ \rho & \rho & \rho & 1 \\ \end{array} } \right]$$

More details about how to estimate $\rho$ can be found elsewhere^31^. The use of this model as an approximation for driver performance has been verified previously^19^. The above model accounts for correlations resulting from multiple observations from the same driver, as is the case for experimental data in this study.

## Results

### Visual status: cluster analysis

**Figure 2:**
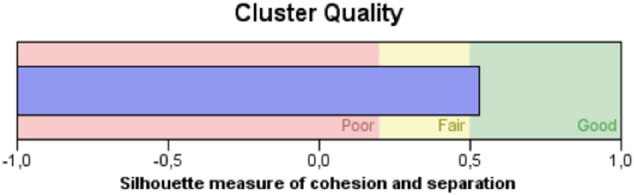
Silhouette measure of cluster quality in terms of visual status.

The two-step cluster analysis identified two groups according to visual status (high and low visual capacity). The silhouette value of cohesion and separation indicated good cluster quality (Fig. 2). Table 3 shows the results in which the entire sample was classified into two similar sized groups based on visual acuity and contrast sensitivity: the low and high visual capacity groups. An unpaired t-test revealed significant differences for visual acuity (t = − 13.473; *p* < 0.001) and contrast sensitivity (t = 4.179; *p* < 0.001).

**Table 3:** Results of a cluster analysis and t-test comparing the two groups identified.

Visual variables	Cluster results		T-test		
	Low visual capacity group	High visual capacity group	t	*df*	*p*
Visual acuity	− 0.01 ± 0.04	− 0.10 ± 0.02	− 13.473	73	< 0.001
Contrast sensitivity	1.80 ± 0.14	1.91 ± 0.09	4.179	65.46	< 0.001
Group size	39 (52%)	36 (48%)	–	–	–

### Speed management across the different driving conditions and road scenarios

Firstly, we conducted a descriptive analysis to compare speed management for the different driving conditions (baseline or texting) and road scenarios. The results are summarised in Table 4.

**Table 4:** Mean ± SD and associated t-test comparing speed management across the different road scenarios under baseline and texting driving conditions.

	Baseline conditions (kph)	Texting conditions (kph)	Mean difference (baseline—distraction)	t	*df*	*p* value
Scenario 1: Dual carriageway, straight, 120 kph SL	− 1.05 ± 11.87	− 17.09 ± 17.46	16.04	8.256	74	< 0.001**
Scenario 2: Dual carriageway, slight bend, 120 kph SL	− 10.70 ± 13.95	− 17.46 ± 15.05	6.76	3.462	74	0.001*
Scenario 3: Mountain, straight, 90 kph SL	− 29.66 ± 13.78	− 38.77 ± 11.52	9.11	4.484	74	< 0.001**
Scenario 4: Mountain, sharp bend, 90 kph SL	− 23.62 ± 9.65	− 31.94 ± 11.18	8.32	5.512	74	< 0.001**
Scenario 5: Mountain, straight, 40 kph SL	2.19 ± 9.26	2.57 ± 9.83	− 0.38	− 0.241	74	0.810
Scenario 6: Mountain, sharp bend, 40 kph SL	− 0.99 ± 6.49	− 2.89 ± 5.65	1.90	2.011	74	0.048*
Scenario 7: Mountain, straight, ascending, 90 kph SL	− 17.41 ± 6.49	− 24.53 ± 12.01	9.02	5.202	74	< 0.001**
Scenario 8: Mountain, straight, descending, 90 kph SL	− 0.98 ± 12.07	− 8.36 ± 15.29	7.38	3.824	74	< 0.001**
Scenario 9: City, straight, parked cars, 50 kph SL	− 17.37 ± 8.28	− 24.66 ± 8.77	7.29	5.980	73	< 0.001**
Scenario 10: City, straight, no parked cars, 50 kph SL	− 8.45 ± 13.58	− 7.30 ± 12.56	− 1.15	− 0.689	73	0.493

*SL* speed limit.

*p < 0.05; **p < 0.001.

Participants’ speed was the furthest below the speed limit along the mountain road sections corresponding to scenarios 3 and 4 where the limit was 90 kph, which indicates that the drivers did not feel as safe driving close to the limit. However, the only time drivers exceeded the speed limit was also on the mountain road (scenario 5), in a straight segment with a 40 kph limit.

On the dual carriageway, they drove more slowly through the slight bend segment (scenario 2) compared to the straight segment (scenario 1), although while distracted they drove at a similar speed for both road geometries (scenarios 1 and 2).

In scenarios 7 and 8, the results showed that drivers reduced their speeds for ascending segments to a greater extent than for descending segments. Likewise, scenarios 9 and 10 evidenced drivers adopted speeds below the limit in urban areas, driving slowest in the segment featuring parked cars (scenario 9).

Differences between the conditions (baseline and texting) were examined using a paired samples t-test. The results, shown in Table 4, indicate drivers generally adapted their speed more under distracted conditions, driving more slowly than the baseline and, therefore, even further below the speed limit. The only scenarios in which drivers did not significantly reduce their speed, and even increased it while texting, were scenarios 5 (mountain road, straight, 40 kph SL) and 10 (city, straight, no parked cars), which could be considered the two simplest segments along the route. On the other hand, mean differences indicated that the driving scenario which elicited the greatest reduction in velocity when driving under texting conditions compared to baseline conditions was scenario 1 (motorway, straight, 120 kph SL).

### Influence of driving conditions, traffic complexity and driver characteristics: generalised linear mixed model (GLMM) results

The GLMM was used to identify possible predictors of speed management. The dependent variable included in the model was speed management and possible predictors were: driving conditions (baseline/texting), road scenario (1–10) and driver characteristics (age, gender, visual status, experience texting while driving and self-perceived increase in risk while texting). To identify whether visual status could predict speed management, visual capacity was introduced as a categorical variable with subjects classified according to the cluster analysis.

The results of the estimates and t-test are shown in Table 5. With respect to driving condition, the model showed that texting while driving was a significant predictor of speed management, as participants drove − 5.08 kph slower while texting WhatsApp messages compared to baseline condition.

**Table 5:** Generalised linear mixed model (GLMM). Estimates of speed management.

Parameter	Coefficient	SE	t-statistic	*p* value	95% CI
**Condition**					
Baseline	–	–	–	–	–
Texting	− 5.08	0.53	− 9.56	< 0.001**	[− 4.04, − 6.12]
**Road scenario/complexity**					
Scenario 1: Dual carriageway, straight, 120 kph SL	0.73	1.59	0.46	0.647	[− 2.41, 3.86]
Scenario 2: Dual carriageway, slight bend, 120 kph SL	− 6.40	1.58	− 4.06	< 0.001**	[− 9.50, − 3.29]
Scenario 3: Mountain, straight, 90 kph SL	− 26.99	1.46	− 18.43	< 0.001**	[− 29.87, − 24.10]
Scenario 4: Mountain, sharp bend, 90 kph SL	− 19.94	1.35	− 14.78	< 0.001**	[− 22,60, − 17.28]
Scenario 5: Mountain, straight, 40 kph SL	10.06	1.34	7.50	< 0.001**	[7.42, 12.70]
Scenario 6: Mountain, sharp bend, 40 kph SL	5.98	1.18	5.07	< 0.001**	[3.65, 8.31]
Scenario 7: Mountain, straight, ascending, 90 kph SL	− 12.82	1.26	− 10.18	< 0.001**	[− 15.30, − 10.34]
Scenario 8: Mountain, straight, descending, 90 kph SL	3.20	1.52	2.11	0.036*	[0.21, 6.19]
Scenario 9: City, straight, parked cars, 50 kph SL	− 13.56	1.28	− 10.56	< 0.001**	[− 16.09, − 11.03]
Scenario 10: City, straight, no parked cars, 50 kph SL	–	–	–	–	–
**Driver characteristics**					
*Age*	− 0.09	0.02	− 3.98	< 0.001**	[− 0.13, − 0.04]
*Gender*					
Male	1.35	0.66	2.05	0.041*	[0.056, 2.65]
Female	–	–	–	–	–
*Visual quality*					
Better	–	–	–	–	–
Worse	0.19	0.55	0.35	0.727	[− 0.89, 1.28]
*Experience texting while driving*^a^					
0-Never	− 1.68	1.04	− 1.61	0.108	[− 3.73, 0.35]
1-1–2 times a year	− 2.43	1.33	− 1.82	0.069	[− 5.05, 0.19]
2-1–2 times a month	− 0.09	1.18	− 0.08	0.937	[− 2.40, 2.22]
3-1–2 times a week	− 3.38	1.24	− 2.71	0.007*	[− 5.82, − 0.93]
4-Daily	–	–	–	–	–
*Self-perceived increase in risk while texting*^b^					
2-Somewhat	9.51	2.32	4.10	< 0.001**	[4.96, 14.08]
3-Quite a lot	1.74	0.78	2.22	0.026*	[0.20, 3.28]
4-A lot	–	–	–	–	–
Intercept	− 6.50	1.58	− 4.12	< 0.001**	[− 9.60, − 3.40]
Number of observations	1500				
AIC	11,343.84				
BIC	11,449.72				

–, Reference category; *p < 0.05; **p < 0.001.

^a^Scale: (0) Never–(4) Daily.

^b^Scale: (0) None–(4) A lot.

Of the different road environments and traffic complexity scenarios, the GLMM results indicated that all the scenarios, except scenario 1, had characteristics that were significant predictors of speed management. Compared to the reference category (scenario 10), the scenario where drivers exhibited the greatest speed management was scenario 3 (mountain, straight road, 90 kph SL), wherein participants drove approximately − 26.99 kph slower. Similarly, the second largest speed reduction (about − 19.94 kph) was effected for scenario 4, which had the same characteristics as scenario 3 but with a curved layout. Although the other two scenarios conducted on mountain roads (5 and 6) were also significant predictors of speed management, the results show that for these segments participants drove at higher speeds than for the reference category (about 10.06 and 5.98 kph respectively).

Regarding the scenarios that included a slope (scenarios 7 and 8), they also proved to be significant predictors of speed management. In this case, the ascending slope was associated with speeds considerably slower than the reference category (− 12.82 kph) but drivers tended to descend at higher speeds than the reference category (3.20 kph). Finally, parked cars in the vicinity when driving in the city was a significant predictor of speed management, with speeds − 13.56 kph slower compared to the reference category.

Driver characteristics were also found to be significant predictors of speed management across the different driving conditions. Participants drove at increasingly lower speeds under the limit (about − 0.09 kph) for every year they increased in age. On the other hand, women drove more slowly than men, with a difference of − 1.35 kph. The results also revealed that experience texting while driving significantly predicted speed management, with drivers who texted daily in their own cars being the fastest group. Self-perceived increase in risk due to texting while driving also predicted speed management. In this case, drivers who felt texting was risky drove at slower speeds. Finally, visual capacity group did not significantly predict speed management.

## Discussion

This study investigated the impact of texting while driving on speed management across different road scenarios with a wide range of features. It also compared driver characteristics, including the influence of visual status, since vision is the main sensory mechanism involved in both driving and the use of smartphone instant messaging applications such as WhatsApp.

### Effect of phone interaction

Our findings show that interacting with the smartphone application WhatsApp while driving had an effect on participants’ speed management. The scenario that caused drivers to reduce their speed the most under texting conditions compared to baseline condition was scenario 1 (motorway, straight, 120 kph SL). This result could be due to the fact that participants received and responded to their first message of the session during this scenario, so they may have acted more cautiously than for the rest of the messages.

According to the GLMM, messaging while driving implies a speed reduction of approximately 5 kph with respect to the baseline session.

Self-regulation behaviours, such as speed reduction, are known to depend on the modality of the phone interaction^1,15,19,32^. Hands-free conversations are the less demanding phone-based distractor, implying only cognitive distraction. However, hands-held conversations add manual distraction and texting combines three types: visual, manual and cognitive distraction. Yet recent meta-analyses and systematic reviews show that hands-free or hand-held mobile phone conversations have a minor effect on crash risk^33-35^, while texting and browsing seem to have a greater effect on driving speed behaviour, leading to reduced speeds^8^. Visual distraction is a key factor in speed reduction, given that drivers must stop looking at the road for considerable periods, leaving them blind to the driving scenario. Along this line, Yannis et al., (2014)^10^ demonstrated mean speed reductions of around 10 and 14 kph when drivers read and wrote SMS, respectively. As in the present case, these speed reductions were greater than those reported in other studies focusing on phone conversations, which illustrates that visual–manual tasks impose a greater demand^36,37^. In our study, the drivers reduced their speed to a lesser degree, maybe because the WhatsApp environment is more familiar considering the revolution this application has brought about in messaging as a means of communication. All our participants were regular WhatsApp users and reported sending at least 30 messages a day. This could give them a greater sense of security while driving compared to writing a text message with other interfaces.

### Effect of driving environment

Driving complexity impacts on the workload required to safely complete the driving task^38,39^, causing self-regulation (or risk compensatory) behaviours among drivers. In our study, participants showed the greatest degree of speed self-regulation (i.e., speed reduction compared to baseline driving conditions) on the mountain road, where the speed limit was 90 kph, which indicates they felt the driving geometry was too complex to drive close to the posted speed limit. This section of the route (mountain road) is considered a relatively complicated one due to its layout and the presence of oncoming traffic.

An analysis of speed management across scenarios showed that, as expected, curved roads require greater adaptation compared to straight roads. Thus, under distracted conditions, they drove through curved segments slower on the motorway (scenarios 1 and 2) and mountain roads (scenarios 5 and 6), by 0.37 and 5.46 kph respectively, compared to the straight segments (Table 4). Previous research has found similar results^13^, suggesting that drivers consider bends to be risky features^18^. Surprisingly, we observed the contrary when comparing scenarios 3 and 4 (mountain road, 90 kph SL), although this could be because the straight section was situated between two sharp curves, hence the configuration may have influenced the result. Additionally, these two scenarios correspond to those with the highest deviation from the speed limit and this may be explained by the fact that the participants were interacting with the message that required the greatest cognitive attention, a simple maths problem^40^.

On the other hand, an ascending slope made drivers reduce their speed in both the baseline and texting sessions. However, when the slope was descending, they practically only drove below the posted speed limit during the texting session. This observation could be because distraction means drivers monitor their speed less and the descending slope causes them to drive more quickly^36^. Finally, our results revealed that participants drove considerably below the posted speed limit when in urban scenarios (9 and 10). Moreover, under distracted conditions, the influence of parked cars in an urban setting resulted in considerable speed adaptations compared to the scenario with no parked cars. Urban scenarios are considered to require the highest workloads given that they are the more visually cluttered. The large amount of information in an urban environment (traffic flow, traffic signals, roundabouts, advertising boards, commercial areas, pedestrians, etc.) means drivers perceive a high load of visual stimuli which they must manage while driving their vehicle. Previous research has also found higher self-regulation of driving speeds while texting in urban scenarios^10,19,41^. The presence of parked cars in the vicinity may also trigger speed adaptations, as there is a sense of greater visual clutter. Parked cars necessitate more interaction with traffic and an increased sense of danger. Indeed, most studies into mobile phone driver distraction have observed changes in speed management when other vehicles were present^20,21^.

### Effect of driver characteristics

Driver age is another significant predictor of speed adaptation, with older drivers reducing their speeds more than younger ones. This result agrees with those published previously in other studies^41^, with older drivers deviating more from the posted speed limit^1,29^. Furthermore, these works highlight that both phone interaction and environment complexity have more pronounced effects on older drivers’ speed behaviour. Research has found that older drivers are better risk estimators^42,43^, possibly because they are aware of a decline in their motor, visual and cognitive capacities, so they try to compensate in more demanding situations. A less widespread result in the literature contrasts with our findings regarding driver age, this is probably due to samples composed uniquely of young drivers (< 30 years old)—in this age range greater experience could lead to drivers adopting faster speeds^19^.

Driver gender was a significant predictor of speed management in our sample. As such, males reduced their speed less than females (they drove 1.35 kph faster). A number of studies have reported that males are more prone to engage in risky behaviours and attitudes during driving such as speeding^44-46^. Women may have less self-confidence regarding their abilities or greater awareness of their limitations, so they perceive risk differently. For example, a study that analysed driving self-regulation in visually impaired older drivers discovered women self-regulated their driving to a greater extent than men^47^. However, despite demonstrating greater caution, the study conducted by Li et al., (2019)^48^ reported that female driving performance during distracted tasks involved more collision risk.

The cluster analysis successfully identified two groups with different visual status (high and low visual capacity). Be that as it may, the GLMM did not identify visual capacity membership to be a significant predictor of driver speed even though both driving and texting WhatsApp messages are strongly dependent on vision. Although, to the best of our knowledge, this is the first time visual status has been included as a possible predictor of speed management under distracted conditions, the influence of vision on driver self-regulation has been explored previously, especially in older drivers. Thus, some studies have found that visually impaired older drivers commonly self-regulate their driving, avoiding challenging situations such as bad weather conditions with poor visibility, rush hour or high-speed roads^26,49^. Our hypothesis was that visual difficulties would increase the workload for both texting and driving tasks, which could make drivers adopt compensatory mechanisms to reduce the risk associated with the increase in visual demand. We expected this behavioural adaptation to be more marked in settings with greater visual clutter such as the urban scenarios included along the route. However, we did not observe this trend, possibly because all the participants had normal vision and a visual acuity above the legal minimum required for driving. Maybe the difference between the two cluster groups is not enough for the participants in the low visual capacity group to perceive themselves as having visual difficulties, so it does not bear an influence on their risk management while driving.

Texting while driving is banned in Spain; nevertheless, a large proportion of the participants admitted they did it quite often (Table 1). In our study, this factor presented a significant association with the drivers’ speed management, as such those who never normally engaged in texting while driving self-regulated their speed more than the rest. What is more, higher scores for self-perceived risk in relation to the dual task correlated significantly with lower speeds. It is expected that dual-task experience should be influenced by safe attitudes towards mobile phone use or self-efficacy^29^. Drivers who confess to daily contact with the application and their mobile phone perceive a higher self-efficacy in the secondary task, as they find typing a quick, straightforward task. These could be the youngest drivers, who sometimes channel a large part of their communication through this type of application^7^. Consequently, drivers who perceive less risk during the dual task exhibit faster speeds^19^.

## Limitations of the study

The findings of this study should be interpreted cautiously due to the limitations of the methods employed. First of all, the use of a driving simulator supposes an important limitation because it cannot provide a truly representative driving environment. Nevertheless, this simulator has been used successfully in a previous study^9^ and there is evidence to support the relative validity of driving simulators with respect to actual driving^50,51^.

On the other hand, messages sent during the trajectory were designed to generate a certain degree of cognitive, manual and visual complexity, but while also maintaining realism insofar as drivers could reply to the message in a real-world situation. However, the differences in the questions sent and the artificial nature of the content could affect the results, so this must be considered when interpreting said results.

Finally, even though our study included a relatively broad sample over a large age range, there are certain aspects that must be taken into account. One is the different distribution in genders and another is the range of WhatsApp usage habits among the participants^7^.

## Conclusions

In our study, we found that speed management is associated with the secondary task, driving environment and driver characteristics. In general, drivers reduce their speeds when faced with more demanding driving situations; while replying to a WhatsApp message and in more complicated situations such as curved roads or with more traffic interactions). Driving speed was also modulated according to driver characteristics such as age or dual task experience and human factors such as self-perceived risk. Nevertheless, our study did not evidence any speed differences between groups with a different visual status, maybe because all the participants had a visual acuity within the legal limit for driving. Future studies should explore speed management in different conditions of visual impairment.

## Data Availability

The datasets generated during the current study are available from the corresponding author on reasonable request.
