# Author Correction: Factors determining speed management during distracted driving (WhatsApp messaging)

**DOI:** 10.1038/s41598-020-79428-2

**Published:** 2020-12-15

**Authors:** Sonia Ortiz-Peregrina, Oscar Oviedo-Trespalacios, Carolina Ortiz, Miriam Casares-Lopez, Carlos Salas, Rosario G. Anera

**Affiliations:** 1 Laboratory of Vision Sciences and Applications, Department of Optics, University of Granada, Edificio Mecenas, Av. Fuentenueva s/n, 18071 Granada, Spain; 2 Centre for Accident Research and Road Safety‑Queensland (CARRS‑Q), Queensland University of Technology (QUT), Brisbane, 4059 Australia

Correction to: *Scientific Reports* 10.1038/s41598-020-70288-4, published online 06 August 2020

This Article contains errors.

Due to a filing error, a previous version of this Article was published.

As a result, the Abstract:

“The objective of this work was to investigate self-regulation behaviours, particularly speed management, under distracted conditions due to WhatsApp use. We also studied the influence of different environments and driver characteristics, introducing visual status as one of them. Seventy-five drivers were evaluated in a simulator study involving two test sessions under baseline and texting conditions. A cluster analysis was used to identify two groups with different visual capacity .Lastly, possible predictors of speed management were studied developing a generalised linear mixed model. Our results show that drivers reduced their speeds in the presence of more demanding driving conditions; while replying to a WhatsApp message, on curved road segments and when parked cars are present. Driving speed also correlated with driver characteristics such as age or dual task experience and human factors such as self-perceived risk. Finally, although there were significant differences in visual capacity between the two groups identified, the model did not identify visual capacity membership as a significant predictor of speed management. This study could provide a better understanding of the mechanisms drivers use when WhatsApp messaging and which environments and driver conditions influence how speed is managed.”

now reads:

“The objective of this work was to investigate self-regulation behaviours, particularly speed management, under distracted conditions due to WhatsApp use. We also studied the influence of different environments and driver characteristics, introducing visual status (i.e., visual acuity and contrast sensitivity) as one of them. Seventy-five drivers were evaluated in a simulator study involving two test sessions under baseline and texting conditions. A cluster analysis was used to identify two groups with different visual capacity. Lastly, possible predictors of speed management were studied developing a generalised linear mixed model. Our results show that drivers reduced their speeds in the presence of more demanding driving conditions; while replying to a WhatsApp message, on curved road segments and when parked cars are present. Driving speed also correlated with driver characteristics such as age or dual task experience and human factors such as self-perceived risk of texting while driving. Finally, although there were significant differences in visual capacity between the two groups identified, the model did not identify visual capacity membership as a significant predictor of speed management. This study could provide a better understanding of the mechanisms drivers use when WhatsApp messaging and which environments and driver conditions influence how speed is managed.”

In the Introduction,

“Research has repeatedly highlighted the negative effects of texting on driving performance^8,9,10^. This driving behaviour doubles the risk of an accident^11^, despite the fact that drivers typically self-regulate their driving when distracted. Self-regulation is a dynamic strategy that drivers use to manage the demands on the resources they require to control the vehicle and perform the secondary task, prioritising the former to minimise the safety risk as much as possible^12^. Self-regulation while distracted includes operations such as paying less attention to the secondary task^12^, over correcting the vehicle’s position^9,13^, and overcorrecting or reducing speed^10,12,13^. Speed reduction is a behaviour commonly observed in all distraction types^8,10,14,15^ because of the difficulties drivers experience in their performance^16^.”

now reads:

“Research has repeatedly highlighted the negative effects of texting on driving performance^8–10^. Naturalistic driving studies have shown that, whereas primarily cognitive secondary tasks do not seem to increase crash risk^50^ manual interactions with a mobile phone significantly increase the risk of an accident, largely due to visual distraction^51^. This result is also supported by a simulator-based study where texting doubled the risk of an accident^11^, despite the fact that drivers self-regulated their driving when distracted. Self-regulation is a dynamic strategy that drivers use to manage the demands on the resources they require to control the vehicle and perform the secondary task, prioritising the former to minimise the safety risk as much as possible^12^. Self-regulation while distracted includes operations such as paying less attention to the secondary task^12^, over correcting the vehicle’s position^9,13^, and overcorrecting or reducing speed^10,12,13^. Speed reduction is a behaviour commonly observed in all distraction types^8,10,14,15^ because of the difficulties drivers experience in their performance^16^. Furthermore, when drivers are distracted by texting messages, their behaviours are different depending on whether they are reading or composing the message. Reading seems to have a greater influence on speed and reaction times, while composing messages affects speed and lane departures, indicating different levels of demand on mental awareness^52^.”

The Methods section has been renamed Data collection. Under the subheading ‘Participants’,

“Seventy-five drivers (19–68 years) were recruited for the study. All were in good general health and did not have any eye diseases. Participants were required to have a binocular visual acuity of 20/40 or better, the legal level for driving in Spain. They must have had a valid driving license for at least one year and driven at least 1000 km in the last year. Likewise, participants were required to be experienced WhatsApp users (≥ 30 WhatsApp messages per day). Table 1 shows the demographic characteristics of the drivers involved in the study.”

now reads:

“Ninety-eight drivers were recruited from the general population via a special online campaign on our website. All were in good general health and did not have any eye diseases. Participants were required to have a binocular visual acuity of 20/40 or better, the legal level for driving in Spain. They must have had a valid driving license for at least one year and driven at least 1000 km in the last year. Likewise, participants were required to be experienced WhatsApp users (≥ 30 WhatsApp messages per day). Of these participants, 16 were excluded due to simulator sickness and seven for not meeting other inclusion criteria (colour vision deficiencies (2), binocular problems (3) and lack of driving experience (2)). Table 1 shows the demographic characteristics of the seventy-five drivers (19–68 years) that were eventually enrolled in the study.”

Under the subheading ‘Visual assessment’, in the subsection ‘Driving simulator: road scenarios’,

“For driving performance data analysis, we selected a representative length of 100 m along each driving scenario.”

now reads:

“For driving performance data analysis, we selected a representative length of 100 m along each driving scenario that contained certain characteristics in terms of road geometry, speed limit and traffic complexity.”

In the subsection ‘Experimental procedure’,

“All participants received at least two training sessions of 15 minutes before the experiment, with a 1-week washout period between them. Then, they were tested in two different sessions to measure driving under baseline and texting conditions.

In the texting condition, participants received six WhatsApp messages, with five short general knowledge questions and one simple mathematical problem. They were instructed to answer these questions in a similar manner as occurs in actual driving, that is, prioritising the driving task. All messages were of a similar length (30–55 characters) and sent at specific points along the route that were strategically selected so drivers could be observed performing the dual task in the 10 scenarios selected for data analysis. Participants drove with the smartphone held by a support located to the right of the steering wheel. They used their own smartphones to ensure they were familiar with its operation.”

now reads:

“All participants received at least two training sessions before the experiment so they could familiarise themselves with the driving simulator. The training sessions lasted about 15 minutes and were conducted using similar routes to those used in experimental sessions, but without any traffic or pedestrians. After completing the training, they were tested in two different sessions to measure driving under baseline and texting conditions. To avoid any possible learning effects, there was a 1 week interval between the training and experimental sessions, and the order of administration of the experimental sessions was also random. If any symptoms of simulator sickness were noted at any stage during the study, the session was interrupted and the participant excluded from the study.

Visual tests were administered at the beginning of experimental sessions, one in each session and in a random order. Participants took a 10-minutes break between the visual test and the experimental drive. Thus, each experimental session lasted about 40 minutes.

In the texting condition, participants received six WhatsApp messages, with five short general knowledge questions and one simple mathematical problem (e.g., “What is the last day of the week?”, “What are the colours of the French flag?”, or “If the bill is €12.50 and I pay with a €50 note, how much change should I receive?”). They were instructed to answer these questions in a similar manner as occurs in actual driving, that is, prioritising the driving task. All messages were of a similar length (30–55 characters) and sent at specific points along the route that were strategically selected so drivers could be observed performing the dual task in the 10 scenarios selected for data analysis. Replies required typing between 2 and 16 characters, as this length is considered realistic from the perspective that a driver could do this in a real driving scenario. During the texting session, we recorded the time and point along the route where drivers started and finished interacting with the mobile phone. This guaranteed that the data analysed in the scenarios corresponded with the moment that participants were engaged in the secondary task. Participants drove with the smartphone in a cradle located to the right of the steering wheel when they were not interacting with it. However, when reading or writing WhatsApp messages, they were free to do so as they would normally, i.e., holding the phone in their hands. This is important to increase the external validity of the experiment, as we wanted to simulate realistic phone use while driving. Participants also used their own smartphones to ensure they were familiar with its operation.”

The Data analysis section has been renamed Method. In the subsection ‘Data analysis and statistical procedures’,

“Data analysis involved two main phases. Firstly, a two-step cluster analysis method was chosen to classify participants into different categories of visual status. This technique assigns participants to a cluster by minimising within-cluster variance and maximising between-cluster variance. The number of clusters is selected using the Akaike information criterion (AIC). The second phase of the study analysed the drivers’ behaviour on different road geometries using a generalised linear mixed model (GLMM) with repeated measures.

The GLMM can be represented as follows^13,30^:

1 $$ g(\mu_{ij} ) = \alpha + {\mathbf{X^{\prime}}}_{i} {{\varvec{\upbeta}}} + {\mathbf{Y^{\prime}}}_{i} {{\varvec{\upgamma}}} + {\mathbf{Z^{\prime}}}_{j} {{\varvec{\uplambda}}} $$

where *g* is the Gaussian link function, **α** is the intercept, **β**, **γ** and **λ** are estimated coefficients of the independent variables. ${\mathbf{X}}_{i}$ is a vector of driver characteristic variables (age, gender, visual status, experience texting while driving and self-perceived risk), ${\mathbf{Y}}_{i}$ is a vector of the driving conditions variable (baseline or texting), and ${\mathbf{Z}}_{j}$ is a vector of variables used to describe the road environment (scenarios 1–10). Coefficients of the link function in the GLMM are estimated from the following equation^13^^,30^:

2 $$ S(\beta ) = \sum\limits_{i = 1}^{k} {\frac{{\partial \mu ^{\prime}_{i} }}{\partial \beta }} V_{i}^{ - 1} (SA_{i} - \mu_{i} (\beta )) = 0 $$

where $V_{i}$ corresponds to an estimation of the covariance matrix of $SA_{i}$ specified as $V_{i} = \phi A_{i}^{1/2} R_{i} (\rho )A_{i}^{1/2}$. Where $A_{i}$ is an $n_{i} \times n_{i}$ diagonal matrix with $v(\mu_{ij} )$ as the *j*th diagonal element. $V_{i}$ varies between drivers, but it can be assumed to have the same form for all drivers. $R_{i} (\rho )$ is an $n_{i} \times n_{i}$ working correlation matrix specified as $\rho$. Constant correlations between any two observations for a given driver are defined as:

3 $$ Corr(SA_{ij} ,SA_{il} )\left\{ {\begin{array}{*{20}c} 1 & {j = l} \\ \rho & {j \ne l} \\ \end{array} e.g.R_{4x4} } \right. = \left[ {\begin{array}{*{20}c} 1 & \rho & \rho & \rho \\ \rho & 1 & \rho & \rho \\ \rho & \rho & 1 & \rho \\ \rho & \rho & \rho & 1 \\ \end{array} } \right] $$

More details about how to estimate $\rho$ can be found elsewhere^31^. The use of this model as an approximation for driver performance has been verified previously^19^. The above model accounts for correlations resulting from multiple observations from the same driver, as is the case for experimental data in this study.”

now reads:

“Data analysis involved two main phases. Firstly, a two-step cluster analysis method was chosen to classify participants into different categories of visual status. This technique assigns participants to a cluster by minimising within-cluster variance and maximising between-cluster variance. The number of clusters is selected using the Akaike information criterion (AIC). The second phase of the study analysed the drivers’ behaviour on different road geometries and of different traffic complexities using a generalised linear mixed model (GLMM) with repeated measures (road scenarios during baseline and texting conditions). We used this model to address the lack of normal distribution of the dependent variable data, i.e., the distribution of the variable “speed management” (Kolmogorov–Smirnov test). Driving conditions (baseline and texting), road scenario/complexity, gender, visual quality group, experience in texting while driving and self-perceived increase in risk in texting while driving were included as factors, and driver age as a covariate.

The use of this model as an approximation for driver performance has been verified previously^19,53^. The above model accounts for correlations resulting from multiple observations from the same driver, as is the case for experimental data in this study.”

In the Results, under the subheading ‘Visual status: cluster analysis’,

“An unpaired t-test revealed significant differences for visual acuity (t =  − 13.473; *p* < 0.001) and contrast sensitivity (t = 4.179; *p* < 0.001).”

now reads:

“An independent t-test revealed significant differences for visual acuity and contrast sensitivity.”

In the Discussion, under the subheading ‘Effect of driver characteristics’,

“The cluster analysis successfully identified two groups with different visual status (high and low visual capacity). Be that as it may, the GLMM did not identify visual capacity membership to be a significant predictor of driver speed even though both driving and texting WhatsApp messages are strongly dependent on vision. Although, to the best of our knowledge, this is the first time visual status has been included as a possible predictor of speed management under distracted conditions, the influence of vision on driver self-regulation has been explored previously, especially in older drivers. Thus, some studies have found that visually impaired older drivers commonly self-regulate their driving, avoiding challenging situations such as bad weather conditions with poor visibility, rush hour or high-speed roads^26,49^. Our hypothesis was that visual difficulties would increase the workload for both texting and driving tasks, which could make drivers adopt compensatory mechanisms to reduce the risk associated with the increase in visual demand. We expected this behavioural adaptation to be more marked in settings with greater visual clutter such as the urban scenarios included along the route. However, we did not observe this trend, possibly because all the participants had normal vision and a visual acuity above the legal minimum required for driving. Maybe the difference between the two cluster groups is not enough for the participants in the low visual capacity group to perceive themselves as having visual difficulties, so it does not bear an influence on their risk management while driving.”

now reads:

“The cluster analysis successfully identified two groups with different visual status (high and low visual capacity). Be that as it may, the GLMM did not identify visual capacity membership to be a significant predictor of driver speed even though both driving and texting WhatsApp messages are strongly dependent on vision. Although, to the best of our knowledge, this is the first time visual status has been included as a possible predictor of speed management under distracted conditions, the influence of vision on driver self-regulation has been explored previously, especially in older drivers. Thus, some studies have found that visually impaired older drivers commonly self-regulate their driving, avoiding challenging situations such as bad weather conditions with poor visibility, rush hour or high-speed roads^26,47^. Our hypothesis was that visual difficulties would increase the workload for both texting and driving tasks, which could make drivers adopt compensatory mechanisms to reduce the risk associated with the increase in visual demand. We expected this behavioural adaptation to be more marked in settings with greater visual clutter such as the urban scenarios included along the route. However, we did not observe this trend, possibly because all the participants had normal vision and a visual acuity above the legal minimum required for driving. Maybe the difference between the two cluster groups is not enough for the participants in the low visual capacity group to perceive themselves as having visual difficulties, so it does not bear an influence on their risk management while driving. There is also the possibility that the simulated environment provides a less complex visual environment than real driving conditions. Although the fidelity of driving simulator environments is becoming more and more realistic, it is still not as varied as in the real world, where we can find a very broad and diverse range of visual information and stimuli. For instance, the simulator used in this study did not include road signs, obstacles and pedestrians with reduced contrast levels, which are common in real driving conditions. Road signs can deteriorate over time and pedestrian clothing can have low levels of saliency. Future studies should include stimuli with different contrast levels in their routes to determine whether visual capacity influences driver behaviour in distracted driving conditions.”

In the section ‘Limitations of the study’,

“The findings of this study should be interpreted cautiously due to the limitations of the methods employed. First of all, the use of a driving simulator supposes an important limitation because it cannot provide a truly representative driving environment. Nevertheless, this simulator has been used successfully in a previous study^9^ and there is evidence to support the relative validity of driving simulators with respect to actual driving^50,51^.

On the other hand, messages sent during the trajectory were designed to generate a certain degree of cognitive, manual and visual complexity, but while also maintaining realism insofar as drivers could reply to the message in a real-world situation. However, the differences in the questions sent and the artificial nature of the content could affect the results, so this must be considered when interpreting said results.”

now reads:

“The findings of this study should be interpreted cautiously due to the limitations of the methods employed. First of all, the use of a driving simulator supposes an important limitation because it cannot provide a truly representative driving environment. Nevertheless, this simulator has been used successfully in a previous study^9^ and there is evidence to support the relative validity of driving simulators with respect to actual driving^48,49^. Also, the order of presentation of the different scenarios during the simulated route was the same in both experimental drives, and this could influence the results in some way. For instance, it is likely that the lower speeds observed in scenario 1 compared to scenario 2 are due to the fact that scenario 1 was the first scenario presented. Scenario 1 could have served as a warn-up period until the participants adapted to the simulator and mobile phone task. Future research and replication are needed in light of potential leaning effects. Nonetheless, it is important to remember that drivers typically experience the same routes in their day-to-day driving. Therefore, this experiment has some level of external validity.

On the other hand, text messages sent during the drive were designed to generate a certain degree of cognitive, manual and visual complexity, while also maintaining realism insofar as drivers could reply to the message in a real-world situation. However, the differences in the questions sent and the artificial nature of the content could affect the results, so this must be considered when interpreting said results. In addition, participants used their own smartphone to ensure that they were familiar with the device. Thus, it is likely that some characteristics of the phone could have differed between participants, e.g., screen size, font size or brightness. Although we cannot control for such small differences, not using their own phone or preferred settings would present potential confounders. A lack of familiarity with the phone could increase the complexity of the phone task, which could trigger self-regulation^54^.”

As a result of the changes above, two References were removed and are listed below:

Wang, X. & Abdel-Aty, M. Temporal and spatial analyses of rear-end crashes at signalized intersections. *Accid. Anal. Prev.* **38**, 1137–1150 (2006).

Liang, K.-Y. & Zeger, S. L. Longitudinal data analysis using generalized linear models. *Biometrika* **73**, 13–22 (1986).

Consequently, References 30–49 were incorrectly listed as References 32–51.

Lastly, an additional five References were omitted. These References are now cited at the relevant points in-text and are listed below:

50. Dingus, T. A. *et al.* The prevalence of and crash risk associated with primarily cognitive secondary tasks*. Saf. Sci*. **119,** 98–105 (2019).

51. Gershon, P. *et al.* Distracted Driving, Visual Inattention, and Crash Risk Among Teenage Drivers. *Am. J. Prev. Med.* **56,** 494–500 (2019).

52. Dimitriou, L., Stylianou, K. & Yannis, G. Capturing the effects of texting on Young drivers behaviour based on copula and Gaussian Mixture Models. *Transp. Res. Part F Traffic Psychol. Behav.* **58,** 930–943 (2018).

53. Choudhary, P. & Velaga, N. Performance Degradation During Sudden Hazardous Events: A Comparative Analysis of Use of a Phone and a Music Player During Driving. *IEEE Trans. Intell. Transp. Syst*. **20,** 4055–4065 (2018).

54. Oviedo-Trespalacios, O., Haque, M. M., King, M. & Washington, S. “Mate! I’m running 10 min late”: An investigation into the self-regulation of mobile phone tasks while driving. *Accid. Anal. Prev*. **122,** 134–142 (2019).

These errors have now been corrected in the HTML and PDF versions of the Article.

